# Acetaminophen’s Role in Autism and ADHD: A Mitochondrial Perspective

**DOI:** 10.3390/ijms26178585

**Published:** 2025-09-03

**Authors:** Stephanie Chu, Seth Woodfin, Emily Bayliss, Merritt Smith, Alan Fulp, Ersilia Mirabelli, William Moore

**Affiliations:** 1 Department of Biology and Chemistry, School of Health Sciences, Liberty University, Lynchburg, VA 24515, USA; sgchu@liberty.edu (S.C.); egbayliss@liberty.edu (E.B.); mlsmith60@liberty.edu (M.S.); afulp@liberty.edu (A.F.); emirabelli@liberty.edu (E.M.); 2 Department of Biomedical Sciences, West Virginia School of Osteopathic Medicine, Lewisburg, WV 24901, USA; swoodfin@osteo.wvsom.edu

**Keywords:** acetaminophen, autism spectrum disorder (ASD), attention-deficit/hyperactivity disorder (ADHD), mitochondrial dysfunction, oxidative stress, N-acetyl-p-benzoquinone imine (NAPQI), prenatal exposure, neuroinflammation

## Abstract

One in 36 children were identified with autism in 2020, a 22% increase from 2018 and a 98% increase from 2010. Simultaneously, attention-deficit/hyperactivity disorder (ADHD) diagnoses increased 36% from 2003 to 2016–2019. Despite this surge, their etiologies remain largely unknown. However, numerous studies document higher incidences of mitochondrial abnormalities in affected individuals. Additionally, acetaminophen has been implicated in these disorders in longitudinal studies and murine models. This paper is a compilation of literature aiming to explore a theoretical framework for acetaminophen-induced mitochondrial damage in utero. It focuses on a toxic metabolite of acetaminophen, N-acetyl-p-benzoquinone imine (NAPQ1), and its role in neuroinflammation. Based on our findings, we recommend further research studying fetal mitochondria after maternal acetaminophen usage.

## 1. Introduction

In December 2023, Judge Denise Cote barred five expert witnesses from testifying that Johnson & Johnson’s Tylenol contributed to the development of autism spectrum disorder (ASD) and attention-deficit/hyperactivity disorder (ADHD) in thousands of children, closing a multi-district litigation [1,2]. Yet, mounting epidemiological evidence suggests an association between high pre-natal and early-life acetaminophen exposure and an increased risk of ASD and ADHD diagnoses [3]. Further, in 2021, a group of 91 doctors and researchers issued a consensus statement that called for precautionary action against the use of the drug during pregnancy [4].

While the etiologies of ASD, often referred to simply as autism, and ADHD remain largely mysterious, the number of cases continues to rise. The Centers for Disease Control and Prevention (CDC) reported that one in 36 children were identified with ASD in 2020, a 22% increase from 2018 and a 98% increase from 2010 [5]. This makes ASD the fastest-growing developmental disorder, affecting more children than acquired immunodeficiency syndrome (AIDS), diabetes, and childhood cancer combined [6,7]. Estimates of the lifetime social cost for an individual with ASD is $3.6 million, and lifetime social costs to date exceed $7 trillion in the United States, expected to reach $11.5–15 trillion by 2029 [8]. Akin to autism rates, ADHD diagnoses have also climbed in the past two decades. In 2016–2019, the CDC revealed that 9.8% of children in the United States were diagnosed with the disorder, a 36% increase from 2003 [9]. Annual societal costs for children and adolescents, due to their ADHD diagnosis, have reached $33.2 billion in the United States, spanning educational and healthcare costs [10]. In 2023, the National Institute of Health contributed $306 million and $72 million to research autism and ADHD, respectively [11].

Both autism and ADHD impose significant fiscal burdens on society and impact the well-being of affected individuals. Nonetheless, a better understanding of the mechanisms of these disorders can pave the way for more efficient and effective treatments and prevention measures.

We present two important disclaimers. First, this paper advocates for the implementation of limits to the usage of acetaminophen by pregnant women, not the abolishment or abandonment of acetaminophen products. Second, the scientific community agrees that autism and ADHD have complex etiologies involving both genetic and environmental factors, and we acknowledge these roles and that acetaminophen can pose a risk to these disorders [12]. This assertion is supported by a review of recent literature implicating the drug, an evaluation of acetaminophen metabolism, and a theoretical framework for acetaminophen-induced autism and ADHD.

## 2. Acetaminophen History

Discovered in the United Kingdom in 1889, acetaminophen (APAP) first entered the market in 1956 and reached yearly sales of 690 million units in the UK just 10 years later [13]. Now, it comprises 8.9% of the global analgesics market with a market value of $9.8 billion [14]. Although Tylenol is a widely recognized brand name for APAP, its exact mechanism of action as an analgesic remains unclear. It was initially believed that it acts through inhibition of cyclooxygenase enzymes, similar to non-steroidal anti-inflammatory drugs (NSAIDs). However, more recent work suggests two other pathways. APAP inhibits the synthesis of 2-arachidonoyl-glycerol (2-AG), an endocannabinoid, possibly in circuits permissive for pain [15]. In addition, researchers discovered that its metabolite, *N*-arachidonoylphenolamine (AM404), acts on sodium channels peripherally [16,17]. APAP is linked to psychological symptoms, such as decreased positive empathy, emotional reactivity, and social pain with regular use, possibly linked to lower physical pain [18,19,20]. It is listed as a Pregnancy Category B substance, indicating that it is generally considered safe for use throughout pregnancy. An estimated 58% of pregnant women in the U.S. use APAP at some point during their pregnancy [21,22].

## 3. Review of Literature Neurodevelopmental Literature

### 3.1. Longitudinal Studies

Despite being considered safe for use during pregnancy, mounting evidence suggests an association between maternal APAP usage and the manifestation of a variety of developmental disorders. In 2021, a group of scientists and doctors published a statement in *Nature Reviews Endocrinology* that urged the Food and Drug Administration (FDA) to review guidelines for maternal use of APAP during pregnancy [4]. In their review, maternal and perinatal use of APAP was strongly associated with neurodevelopmental disorders, including ASD and ADHD, in 26 out of 29 observational studies. These studies collected data from 220,000 mother-child pairs, spanning multiple regions worldwide. Beyond neurodevelopmental disorders, the article revealed that APAP was correlated with significant abnormalities in motor and reproductive development.

Despite these data, the FDA and the Society for Maternal Fetal Medicine (SMFM) have not made any changes or comments regarding the medicinal status of APAP [23,24]. In the past, the SMFM cited three limitations of the aforementioned research. These studies were deemed insufficient to warrant changes because they: (1) relied on mothers’ self-reported APAP usage, (2) lacked precise quantification of APAP dosage, and (3) frequently utilized questionnaire-based methodologies.

Prior to 2019, no studies adequately addressed these limitations. However, at Johns Hopkins University, researchers met these standards by evaluating members of the Boston Birth Cohort [3]. They analyzed biological samples of 996 mother-child dyads, focusing on the presence of acetaminophen and its byproducts in umbilical cord blood samples, dividing them into groups of low, medium, and high exposure. Maternal age, ethnicity, stress, alcohol and illicit drug usage, education, body mass index (BMI), fever during pregnancy, and other covariates were controlled during the study. Their findings revealed a notable association between in utero acetaminophen exposure and an increased risk of ADHD and ASD in childhood. Additionally, children in the high-exposure group were 3.62 times more likely to be diagnosed with autism and 2.86 times more likely to be diagnosed with ADHD compared to the low-exposure group. One limitation of this study is that cord samples were collected at birth, which may not be representative of overall exposure. Nonetheless, the perinatal period is a markedly critical period for development of behavioral abnormalities in animal studies [25]. By using biological samples, this study fulfills the criteria set by SMFM [26].

A second foundational study was published in 2020 using acetaminophen levels in meconium, the first stools of newborns [27]. The meconium reflects the accumulation of drug exposure in neonates for the last two-thirds of pregnancy [28]. Meconium samples were collected from 345 children of the Canadian Birth Cohort, and their APAP contents were quantified [27]. At ages six and seven, it was determined whether a child had received an ADHD diagnosis. When children reached ages 9–11, magnetic resonance imaging (MRI) scans were conducted to assess brain connectivity in three networks associated with ADHD: (1) salience/cingulo-opercular, (2) central executive/frontoparietal, and (3) the default mode networks. Only 48 children were included in the MRI results as this step was ongoing at the time of publication. The results showed that, compared to those with no APAP exposure, infants with high exposure were found to be 4.1 times as likely to be diagnosed with ADHD. Furthermore, those with detectable APAP levels in meconium had decreased connectivity in the three networks, with lower levels of connectivity correlating with more pronounced symptoms of ADHD.

Not all studies came to this conclusion. In 2024, a large Swedish birth cohort was studied for a potential causal association between APAP and ASD/ADHD [29]. Researchers primarily measured exposure based on whether mothers used any acetaminophen in pregnancy. Their secondary exposure metric was obtained via quantification of APAP dosage in prescription dispensations during pregnancy. Of the nearly 2.5 million children in the cohort, 185,909 were exposed to APAP. The prevalence of autism among children without APAP exposure to those with exposure was 1.33% and 1.55% respectively with a hazard ratio of 1.05. Likewise, the rate of ADHD among children without APAP exposure to those with exposure was 2.46% and 2.87% respectively with a hazard ratio of 1.07. To put this in perspective, the hazard ratio of a child developing neuropsychiatric disorders because of long term prenatal opioid exposure of 60 days or more is 1.95 [30]. Nonetheless, researchers used a sibling control analysis to account for familial confounders, such as parents’ autistic traits. One potential downside of sibling comparison studies is that they eliminate potential mediators shared between families that interact with acetaminophen [31]. No association between APAP exposure and ASD/ADHD was found with the analysis.

However, this study has two limitations that impact its ability to quantify APAP dosage [29]. First, the primary exposure metric in this study was the ever-use of acetaminophen. This neglects to quantify dosage. Second, because its secondary exposure metric quantified APAP use through prescription dispensations, it does not account for over-the-counter APAP use. Fifty-four percent of pregnant women use over-the-counter acetaminophen, according to one Iowa-based study [32]. Further, past research shows that prescription dispensations do not always reflect actual use. The mean implementation adherence among pregnant women was 72% in one study, and another study found that prescription guidelines compliance among Danish pregnant women was 43% [33,34]. With these two limitations, the study cannot accurately estimate APAP usage. As previously mentioned, longitudinal studies must quantify APAP usage since a critical point of exposure may exist [23,24,29] (Table 1). Although this study explores confounders through sibling control analyses, the lack of APAP dose quantification hampers its ability to establish the presence of absence of a correlation between the two variables.

Other studies have utilized sibling control analyses and came to varying conclusions. One found that maternal APAP usage for over 28 days correlated with ASD/ADHD but found no correlation with a sibling control analyses [35]**.** Another study also found that maternal APAP usage for over 28 days correlated with ASD/ADHD in a three-year follow up, confirmed by the sibling control analyses [36] (Table 1).

#### Genetics Confounders

The research we have shown presents a strong association between maternal acetaminophen intake and adverse offspring neurodevelopmental outcomes. Yet, some opine that maternal genetics pose a potential confounding factor if it causes both increased maternal acetaminophen usage (e.g., due to frequent fevers in pregnancy) and offspring neurodevelopmental outcomes. In that case, there would be merely a correlation between acetaminophen exposure and ASD/ADHD diagnoses, not causation. The Swedish birth cohort study indicated that parental traits associated with ASD/ADHD are related to increased use of acetaminophen [29]. For instance, the Avon Longitudinal Study of Parents and Children cited that maternal ADHD polygenic risk scores correlate with lower age at delivery, higher BMI, increased odds of smoking, and increased odds of using acetaminophen [38]. In other words, mothers with higher genetic predispositions for ADHD are also more likely to exhibit these distinct traits. Some argue that these traits do not independently cause neurodevelopment outcomes; rather, genetics underlie both the traits and the disorders.

However, genetic factors alone account for only 10–20% of ASD cases, and concordance rates among monozygotic twins are incomplete [39]. This highlights the role of environmental influences alongside genetic predisposition, further supporting the argument that acetaminophen may act as a potential environmental risk factor. Further, the listed studies have controls for these covariates. The first study, which used umbilical cords, accounted for parental neurodevelopmental disorder diagnosis, age at delivery, smoking status, and BMI, among other covariates [3]. Additionally, the meconium study controlled for all but the first covariate, analyzing it in a separate test [27]. Adjusting for maternal ADHD diagnoses resulted in the data shifting by 2% in confirmation of the hypothesis. It is important to note that diagnoses and polygenic risk scores are not the same. While polygenetic risk scores estimate a person’s inherited genetic susceptibility based on multiple common genetic variants, a clinical diagnosis reflects observable symptoms and behaviors, which may be influenced by both genetic and environmental influences. Still, these findings indicate that genetics alone are not the sole cause of the development of ASD and ADHD.

### 3.2. Murine Studies

While sibling control analyses are used to remove genetic confounders, another method uses randomized mouse studies to determine causation [35,36]. In these studies, pregnant or neonate mice are given doses equivalent to or in excess of recommended dosage for pregnant women. Newborn pups up to postnatal day 10 are often used to represent human fetuses based on the neurodevelopmental milestones of mice [24]. Control mice are often given a saline injection instead. The offspring are then studied for behavioral or biochemical variations.

#### 3.2.1. Behavioral Results

There is a plethora of methods to study behavior in mice. Current research methods include counting pups’ ultra-sound vocalizations during maternal separation, total locomotion and motor behaviors, activity trends in an unfamiliar environment, maze learning, and pain responsiveness [37,40]. Using these metrics, researchers found that exposure to acetaminophen on embryonic days 4–10 caused increased vocalizations and decreased ambulation, which is a behavior associated with anxiety [37]. Pups given acetaminophen on postnatal day 10 increased errors and time to completion for mazes in adulthood, which is related to decreased spatial working memory [40]. Researchers further observed that adult mice had decreased activity for the first 20 min in an unfamiliar environment and increased activity in the last 20 min compared to controls when treated with acetaminophen on postnatal days 3, 10, and 19 [25]. These findings are associated with delayed habituation, which is one of the key features observed in neurodevelopment disorders such as ASD [41]. Lastly, adult mice exposed to acetaminophen on postnatal day 10 responded less to the analgesic effects of a new dose [40]. Adult mice with no previous exposure, low exposure, and high exposure to acetaminophen were given a dose 30 min prior to being placed on a hot plate. Mice without prior exposure exhibited a delayed response to the painful stimulus, indicating the efficacy of the drug’s analgesic properties. However, mice with the highest previous exposure to APAP displayed the quickest signs of pain, indicating lasting decreased sensitivity to the drug. This study concluded that APAP disrupts cognitive function and impacts adult sensitivity to APAP and its analgesic and anxiolytic properties.

#### 3.2.2. Biochemical Results

A few studies display the biochemical impact of early APAP exposure in mice. One study measured the presence of brain-derived neurotrophic factor (BDNF) in mice exposed to APAP on postnatal day 10 compared to controls [36]. BDNF supports axonal growth, neuronal survival, formation of synapses and cell migration. APAP-treated mice exhibited 183% of the BDNF level of control mice in the frontal cortex and 26% of the BDNF level in the parietal cortex [40,42,43]. In another study, researchers used RNA sequencing to determine distinct gene regulation in the prefrontal cortex of treated pups [37]. Upregulated genes were enriched in pathways related to the DNA damage response and glutathione metabolism, implying oxidative stress-induced DNA damage. Further, immune-mediated pathways were also upregulated, which could link to impaired neurodevelopment. Histology, hormone levels, and other developmental metrics were also found to be impacted by a single APAP administration slightly above the recommended human dose equivalent [44]. It was found that pregnant mice exposed to APAP showed signs of liver toxicity, affected immune adaptation to pregnancy, harm to placental functional regions, decreased maternal progesterone, hindered fetal development, lower fetal weight, and decreased T-cell development. These studies illustrate the many facets of biochemical damage induced by APAP exposure in mice.

While numerous studies have established a correlation between maternal acetaminophen use and ASD/ADHD, few agree on a proposed mechanism. The next section aims to explore a theoretical framework for acetaminophen-induced ASD/ADHD. However, we must first explain the association of autism and ADHD, reviewing literature on these disorders’ profiles.

## 4. Dimensional Theory of ASD and ADHD

Approximately 20–80% of children diagnosed with ASD also meet the diagnostic criteria for ADHD, while 30–60% of those diagnosed with ADHD display significant symptoms of autism [45]. Although traditionally viewed as distinct, the high co-occurrence of ASD and ADHD has prompted increasing interest in the possibility of a shared etiology [46]. The evidence for this theory falls under three categories: statistical analysis, common genetics, and shared medical conditions.

### 4.1. Statistical Analysis

#### 4.1.1. ASD Diagnostic Criteria

Autism is a complex neurological and developmental disorder. The term ‘autism’ is derived from the Greek word ‘autos,’ meaning self, reflecting the tendency of individuals with autism to appear self-absorbed [47]. Individuals with ASD are characterized by two major symptoms: difficulties in communication and social interaction, and restrictive, repetitive interests and behaviors. Additionally, they often exhibit hyperfocus on specific interests while struggling to filter out distractions [48]. ASD frequently co-occurs with several other psychiatric disorders, such as ADHD, obsessive–compulsive disorder, anxiety, and aggression [49]. Furthermore, gastrointestinal and musculoskeletal disorders are commonly associated with ASD [50].

There is a sizeable range of severity in this disorder, so many refer to it as a spectrum. For instance, certain individuals may have more intense symptoms that require significant intervention and aid, while others can function well independently [9].

#### 4.1.2. ADHD Diagnostic Criteria

The *Diagnostic and Statistical Manual of Mental Disorders, 5th Ed*. (DSM-5) outlines the diagnostic criteria for ADHD, which include inattention, hyperactivity, and impulsivity. Hallmarks of ADHD include excessive talking, fidgeting, carelessness, and avoidance of tasks requiring sustained mental effort. An individual must exhibit at least six specific traits to be diagnosed with the disorder [51]. However, critics contend that the term ‘attention deficit’ is misleading, as individuals with ADHD can exhibit intense focus on certain tasks. Dr. Edward Hallowell, a foremost expert on ADHD, describes it as “…having a Ferrari engine for a brain with bicycle brakes. Strengthen the brakes and you have a champion” [52]. While the two former descriptions may seem contradictory, there is a common line of reasoning between the two. Dr. Hallowell explains that apparent inattentiveness may not stem from a lack of capacity; rather, individuals with ADHD may exhibit difficulty regulating attention toward socially or contextually appropriate targets [52].

At first glance, the diagnostic criteria for these disorders seem to have no commonalities. ASD symptoms often align with introversion and overstimulation, leading to avoidance of social interactions, while symptoms of ADHD may suggest a lack of social inhibition. However, one underlying symptom has intrigued researchers. Both ASD and ADHD groups struggle with inattention. This similarity led previous DSM versions to exclude dual diagnoses of ASD and ADHD [53].

Attention is defined as “a process of selection applied to the product of perception and may even be directed toward memories” [53]. In other words, attention refers to any choice to focus on ideas or memories. It involves filtering information and can recruit working memory, particularly when the object of attention is represented internally in contrast to an external stimulus. Attention relies on various executive functions [53].

#### 4.1.3. Random Forest Classification and Community Detection

Proponents of the continuum theory suggest that ASD and ADHD lie on a spectrum, with ASD being more severe. According to this theory, all individuals with ASD should exhibit ADHD symptoms [45]. However, statistical analysis suggests a more complex explanation than a single, linear continuum. A dimensional approach is more likely. Researchers used random forest classification on questionnaire data from 219 children aged 7–14 years, including participants who are neurotypical and those with autism and/or ADHD. The statistical analysis could not accurately determine the participants’ diagnoses based on the data. This suggests that the characteristics of these disorders are statistically difficult to distinguish. They also used a community detection approach to group individuals based on symptoms. The analysis identified four distinct categories, none of which were specifically ADHD or ASD. In fact, the categories span across diagnoses. Researchers also used taxometric analysis to determine whether the groups were dimensionally different or categorically distinct, which was dependent on the comparison curve fit index falling below or above 0.5. Individuals with ASD were categorically distinct from neurotypical children, but those with ASD and ADHD were only dimensionally distinct. Moderate ASD symptoms did not reliably correlate with high ADHD symptoms, challenging the continuum hypothesis and favoring the dimensional model instead [46].

### 4.2. Common Genetics

Environmental and genetic factors likely interact in the etiology of ASD and ADHD [12]. Genetic influence is particularly strong with restricted/repetitive behaviors and hyperactivity/impulsivity. One study found 17 loci that ASD and ADHD have in common [54]. Another study examined genome-wide association studies and found that 35.7% of single-nucleotide polymorphisms (SNPs) associated with ASD colocalized with ADHD SNPs. The study also determined that 19.6% of ADHD SNPs colocalized with ASD SNPs [55]. These overlapping loci suggest partially convergent genetic architectures.

### 4.3. Shared Medical Conditions

ASD and ADHD also share several comorbid medical conditions, including joint hypermobility, sleep disorders, and gastrointestinal dysfunction. Of particular relevance are shared heavy metal and immune biomarkers, which could indicate a common physiological vulnerability [56,57,58]. Since these medical conditions affect several high-energy systems (nervous, immune, and digestive systems), researchers speculate that both disorders may originate in the disruption of cellular energy production processes [59,60]. Within this framework, the etiologies of these disorders become clearer. Given their central role in cellular energy metabolism, mitochondria are likely involved and will be discussed in more detail later in this paper.

## 5. Etiologies

### 5.1. APAP in Utero

**Figure 1:**
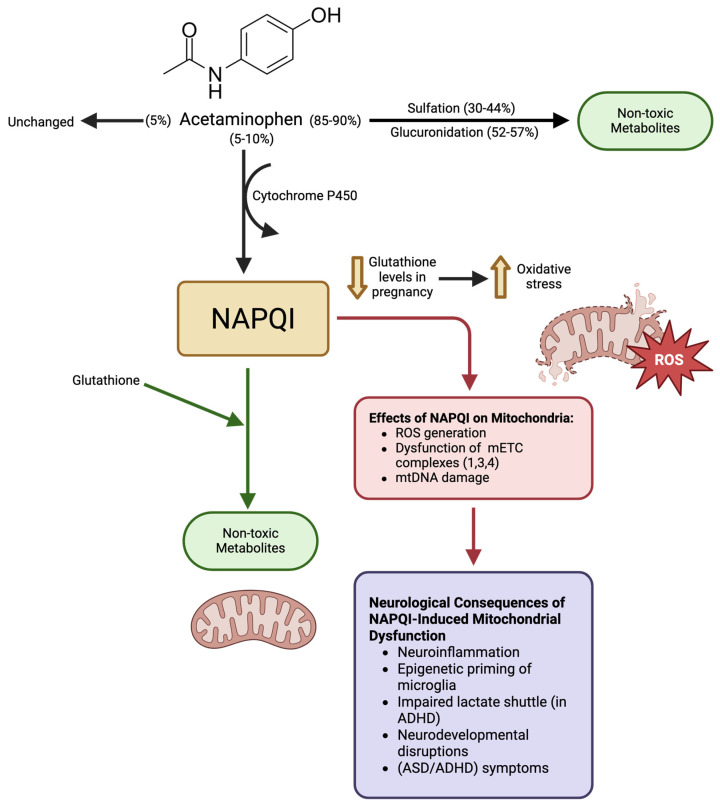
Acetaminophen metabolism and NAPQI-mediated mitochondrial effects leading to neurodevelopmental outcomes. Most acetaminophen is detoxified via sulfation and glucuronidation to form non-toxic metabolites (black arrows). A smaller fraction (5–10%) is metabolized by cytochrome P450 to NAPQI, which is normally neutralized by glutathione and converted to non-toxic conjugates (green arrows). During pregnancy, however, reduced glutathione levels increase vulnerability to NAPQI, which can result in mitochondrial oxidative stress, elevated ROS production, disruption of electron transport chain complexes I, III, and IV, and mitochondrial DNA damage (red arrow ending in red box). These effects on mitochondria contribute to neuroinflammation, epigenetic priming of microglia, impaired astrocyte-neuron lactate shuttling (relevant to ADHD), and neurodevelopmental disruptions that may manifest as ASD and ADHD symptoms (red arrow ending in a purple box). The image was generated by using BioRender.com.

To understand how APAP threatens cellular energy production, we must examine its metabolism. For adults, the recommended maximum daily dose is 1000 mg. Acute doses exceeding 4000 mg or chronic maximal usage can cause severe liver damage [61]. Approximately 90% of a dose is excreted in the urine after conjugation with sulfate (30–44%) and glucuronide (52–57%). Another 5% is excreted unchanged. Cytochrome P450 converts the remaining 5–10% to N-acetyl-p-benzoquinone imine (NAPQI) (Figure 1) [62]. This metabolite is unstable and may facilitate an increase in toxic reactive oxygen species (ROS). NAPQI is typically neutralized by binding to the antioxidant, glutathione (Figure 1) [62]. Given that mitochondria are both major ROS producers and highly sensitive to oxidative damage, they rely on a robust antioxidant defense system [63]. However, excessive doses deplete glutathione stores allowing NAPQI to damage the cell, particularly mitochondria [64,65].

NAPQI forms adducts with mitochondrial proteins, resulting in deformations of ATP synthase, mitochondrial electron transport chain complexes, and mitochondrial DNA (mtDNA) [66,67,68]. Timely administration of N-acetylcysteine can replenish glutathione and reverse this damage, but without intervention, oxidative stress activates redox kinases [69]. One such kinase, c-Jun N-terminal kinase, translocates into mitochondria, and exacerbates oxidative stress [70]. This opens the mitochondrial membrane-permeability transition pore and disrupts mitochondrial membrane potential, inducing programmed necrosis and liver failure. Given the lethality of NAPQI, it is clear why APAP is scrutinized.

In the United States, acetaminophen toxicity causes 5600 emergency department visits, 2600 hospitalizations, and 500 deaths annually [61]. Approximately half of these fatalities are accidental, and APAP overdose is the leading cause of liver transplantation in the country.

Although the liver, with its high mitochondrial content, is the primary organ affected by acetaminophen poisoning, other high-energy organs are also at risk with regular APAP use. Recent evidence shows that regular therapeutic doses of APAP cause cardiomyocyte mitochondrial dysfunction and decreased cell viability. This increases the risk of hypertension and heart disease [71].

### 5.2. Vulnerable Populations: Fetuses and Infants

APAP is considered one of the few safe drugs for fever reduction during pregnancy, which is critical to the health and safety of the mother and fetus [72]. However, in utero and neonatal stages are key developmental periods with increased susceptibility to environmental toxins [73]. Additionally, physiological changes during pregnancy impact drug metabolism, necessitating greater caution when administering medicines to pregnant mothers.

While glutathione typically counters the effects of NAPQI, pregnancy reduces glutathione levels (Figure 1). This is due to oxidative stress, which is characterized by increased free-radicals and impaired antioxidant capacity. Serum glutathione levels can fall by as much as 26% in pregnant women compared to non-pregnant women, with an 80% decrease between the first to second trimester [74,75]. Thus, any increase in oxidative burden poses a greater risk to pregnant women and their fetuses. Maternal inflammation triggers the release of cytokines and oxidative stress molecules that cross into the fetal environment. Because the fetus’ immune and nervous systems are still in development during gestation (and afterwards), it is susceptible to potentially harmful effects of such molecules crossing via the placenta [76].

In addition, these lasting effects can be attributed to maternal inflammation altering the epigenetic machinery of fetal cells and priming the microglia that, as a result, become more responsive to possible stressors afterwards. Microglia are resident immune cells of the CNS and are formed during early development and maintained throughout life, therefore maternal inflammation can lead to life-long impact on the individual’s neuroinflammatory responses [77]. Further, unprocessed APAP has been shown to cross the placental barrier and remain in fetal circulation for extended periods, although only a small portion is converted to NAPQI once there [3,78].

### 5.3. Inflammation in the Mitochondria

A growing body of evidence highlights mitochondrial dysfunction in ASD and ADHD [79,80]. With symptoms affecting high-energy systems, including central nervous, musculoskeletal, and gastrointestinal systems, many believe these disorders stem from cellular metabolism issues. First observed in 1985, there have since been 252 studies investigating the association between mitochondrial abnormalities and ASD symptoms [81,82]. More recently, hundreds of articles have studied mitochondrial implications in ADHD pathogenesis [80].

One key driver of this dysfunction is prolonged oxidative stress, which may begin with maternal inflammation, triggering a cascade of fetal inflammation. Fetal inflammation increases ROS production in the mitochondria, which can potentially lead to neuroinflammation, neuronal development dysfunction, and ASD [83,84]. Notably, developmental neurotoxins that induce oxidative stress are linked to ASD, ADHD, and schizophrenia [63,85]. Past authors have concluded that maternal usage of APAP can induce oxidative stress and ASD in [86,87]. Here, we explore that framework in mitochondria.

#### 5.3.1. Biomarkers of Oxidative Stress in ASD

Mitochondria from individuals with ASD demonstrate a more pronounced response to acute ROS exposure in lymphoblastoid cell lines compared to controls. This exaggerated response is believed to be due to mitoplasticity, a cellular adaptation to prior oxidative damage [88]. Supporting this, individuals with ASD have often been shown to exhibit low total antioxidant capacity and elevated oxidative stress markers, indicating a chronic redox imbalance [89].

Up to 80% of individuals with autism show biomarkers for mitochondrial dysfunction [84,88]. These markers encompass a range of biochemical abnormalities, such as altered ratios of mtDNA to nuclear DNA, and aberrant expression of mitochondrial inner membrane and matrix proteins [81,90]. Both direct markers, including pyruvate, lactate, lactate-to-pyruvate ratio, alanine, alanine-to-lysine ratio, acyl-carnitines, and ubiquinone, and indirect markers, such as ammonia, carnitine, creatine kinase, alanine aminotransferase, and aspartate aminotransferase, have been used to identify these dysfunctions [91]. In particular, elevated blood lactate and pyruvate levels have been reported in up to 76% of individuals with autism [92]. Since both lactate and pyruvate are substrates for ATP production, their prominent levels suggest impaired oxidative phosphorylation and inefficient energy production. The presence of these metabolic imbalances further underscores the role of mitochondrial dysfunction as a potential contributing factor to the pathophysiology of ASD [91].

In addition, mtDNA variations are common for those with ASD. Mitochondria are unique in that they have their own genetic material, encoding mitochondrial proteins and all the electron transport chain proteins except complex II [93]. Complex II is encoded in autosomal DNA, or nDNA. mtDNA is 10 times more vulnerable to mutations and oxidative stress than nDNA due to a lack of histones protection, fewer repair enzymes, and increased susceptibility to supercoiling damage [94]. One study reported that 16.6% of ASD participants had mtDNA deletions, and 90% of those with deletions had rare single-nucleotide variations in ASD-related mtDNA genes [79].

Lastly, in addition to abnormal biochemical markers and mtDNA deletions, ASD patients exhibit variable levels of key mitochondrial proteins. One study found that 88% of patients with autism had deficiencies in the electron transport chain complexes I, III, and/or IV [92,95]. This vulnerability is partly explained by the fact that complexes I and III, both major ROS producers, contain subunits encoded by mtDNA, making them more susceptible to oxidative stress; likewise, complex II, transcribed solely from nDNA, confers its own resistance from mtDNA damage [63,93]. Thus, dysfunctions in complexes I, III, and IV are more likely to occur under oxidative stress. As cells from individuals with autism adapt to oxidative stress, lower activity of complexes I and III may become an adaptive feature. While initially a consequence of oxidative damage, this downregulation appears to reflect a protective response aimed at minimizing further ROS generation.

Mitochondrial function in ASD murine models verifies this explanation. The BTBR mouse strain, developed as a preclinical model for ASD, displays decreased mitochondrial oxygen consumption and behavioral abnormalities that parallel core ASD symptoms. Mitochondrial replacement in BTBR mice reduced ASD symptoms [95]. The ketogenic diet, by shifting cellular energy metabolism toward fatty acid oxidation via complex II, appears to compensate for impaired complex I activity and supports healthier mitochondrial function [96].

#### 5.3.2. Biomarkers of Oxidative Stress in ADHD

Aberrant mitochondrial biomarkers are also evident in ADHD patients. In one study, researchers created cybrids from the SH-SY5Y neuroblastoma cell line by using platelets from individuals with ADHD. The researchers transferred mitochondria from platelets of individuals with ADHD to a mitochondria-less neuronal stem cell line [60]. The resulting cells displayed oxidative imbalance, reduced cellular and mitochondrial respiration rates, lower ATPase transcript levels, decreased mitochondrial membrane potential, and impaired mitochondrial complex activity. These findings suggest that mitochondrial abnormalities in ADHD are intrinsic to the organelle itself rather than the nuclear genome. In support, clinical data show that children with ADHD have an estimated 1.3-fold increase in mtDNA copy number compared to neurotypical controls [97], which may reflect a compensatory response to mitochondrial dysfunction.

One proposed mechanism for abnormal processing in ADHD that is gaining interest, is termed the lactate shuttle hypothesis. This perspective suggests that cellular energy metabolism is disrupted due to deficiencies in lactate transportation and/or production between astrocytes and neurons [98,99].

Within the nervous tissue, lactate was previously believed to be an unnecessary or even toxic substrate [100]. Lactate is a byproduct of anaerobic respiration, which is much less efficient compared to aerobic respiration. In anaerobic respiration, a net of two molecules of ATP are produced from the metabolism of every glucose molecule. However, aerobic respiration produces 30–32 molecules of ATP for the same amount of glucose [100]. In muscle tissue, lactic acid builds up after strenuous exercise due to insufficient supplies of oxygen [101]. Recent research has found lactate to be a useful and necessary component of cell metabolism, especially in the brain [100].

Indeed, lactate acts as an energy source for neurons and serves in signaling pathways [101]. Its production and reception involve two key players: astrocytes and norepinephrine-releasing neurons. Once produced in the astrocyte, lactate is shuttled to the neuron via transporters and converted for use in aerobic respiration. Hence, this process is termed the ‘lactate shuttle’ [100].

Neural activity stimulates astrocytes to produce lactate from glucose through glycolysis [99]. Astrocytes are best suited for this role due to their ability to store glucose as glycogen and their simultaneous connection to many neurons [102]. Once produced, lactate is transported from the astrocyte through monocarboxylate transporters [100]. Lactate is then converted to pyruvate by lactate dehydrogenase, enlisting neuronal mitochondria for use in aerobic respiration. Neurons produce ATP through aerobic respiration, primarily consumed in restoring membrane potential. The conversion of lactate to pyruvate additionally produces NADH, a high-energy molecule, from NAD^+^. Because of the change in charge, the redox state of the neuron is altered, enhancing N-methyl-D-aspartate (NMDA) receptor activity. Positive modulation of NMDA receptors results in greater calcium currents. This, in turn, activates intracellular signaling cascades, which can trigger the release of norepinephrine via calcium-dependent pathways linking energy metabolism with neurotransmitter regulation [100].

These lactate-induced signaling cascades enable plasticity, memory consolidation, and excitability, particularly in neurons triggered by norepinephrine [100]. Several genes involved in plasticity are activated by the NMDA cascade, including BDNF and early growth response protein 1 [103]. In rat studies, inhibiting lactate production in astrocytes also inhibited in vivo hippocampal long-term potentiation. However, injecting lactate and reactivating its conversion in neurons reversed these effects. Spatial memory and memory consolidation have also been shown to require lactate. Lastly, lactate is sufficient for neuron excitability. Applying intracellular lactate to isolated pyramidal and subthalamic nucleus neurons resulted in depolarization, while inhibiting lactate conversion in astrocytes caused hyperpolarization in the same neurons [104]. Many studies show the necessity of lactate for the integrity and support of excitatory synaptic transmission, especially during sustained neuronal activity [98]. It is important to note that lactate itself is not the direct ligand for NMDA receptors. Rather, NMDA receptors, one of the “big three” ionotropic glutamate receptors and a principal mediator of excitatory neurotransmission in the CNS, are activated by glutamate. The role of lactate is to modulate neural redox state and energy metabolism, which then serves to indirectly enhance NMDA receptor activity, thus facilitating downstream calcium signaling and synaptic plasticity [100,103,105].

In the case of ADHD, this system fails, and researchers posit that structure is not the issue at all. Insufficient energy supplies lead to ineffective or limited neural functioning [98]. Indeed, certain regions of the brain, including the prefrontal cortex, in ADHD patients consume less glucose than controls [106]. This area is implicated in ASD and ADHD due to its role in cognitive, behavioral, and executive functions [107]. In fact, positron emission tomography reveals a global 8% decrease in brain energy utilization across half (*n* = 60) of the evaluated brain regions [108]. This could imply that glucose is not efficiently converted to lactate in the astrocytes.

As mentioned previously, the lactate shuttle is utilized by norepinephrine-releasing neurons located in the locus coeruleus among other neuronal populations. The locus coeruleus is deemed necessary for optimal attention performance [109]. The locus coeruleus modulates widespread cortical and subcortical regions by releasing norepinephrine, which influences neuronal metabolism and synaptic activity. During periods of heightened neural activity, locus coeruleus neurons initially rely on glucose-derived energy for the first 5–12 s, then turn to lactate use. Norepinephrine plays an important role in this transition by enhancing astrocytic glycogenolysis and lactate production, which effectively supports the energy demand of attentive states [98].

Thus, if lactate is in short supply, due to disruptions in the lactate shuttle or impaired astrocyte neuron metabolic coupling, a neuron will quickly deplete its energy stores. Maintaining attention becomes difficult due to neural fatigue, which aligns with the attention difficulties of ADHD [98,110]. While this may seem contradictory to ADHD hyperactivity, it has been described as a response to seeking new stimuli once attention fades [98]. Thus, it is posited that patients with ADHD suffer from inadequate supplies of lactate due to impairments in the norepinephrine-regulated lactate shuttling process.

Further, brain intake of lactate from the blood stream is higher in rat models of ADHD [111]. In these models an increased expression of lactate transporters across the blood–brain barrier was observed compared to controls, indicating that the brain can compensate for intrinsic deficits by using other bodily sources. These researchers also posited that hyperactivity is a mechanism for individuals with ADHD to build up lactate stores for brain shortages. In support of this notion, exercise has been found to reduce ADHD symptoms and increase lactate usage in the brain in general [111,112].

## 6. Connective Tissue Symptoms

In addition to atypical behavior, ASD and ADHD patients frequently possess gait abnormalities, such as toe-walking, and have significantly greater joint mobility than those without these disorders [113]. In one study, researchers found that patients with ASD, ADHD, and/or Tourette Syndrome had a five times greater prevalence of hypermobility than the control group [57]. Other studies have found that 73% of children with autism aged five and older present with joint hypermobility and walk 1.6 months later than their peers without autism [113,114]. This likely relates to articular cartilage malformation.

Articular cartilage is formed in the embryonic stage and is vulnerable to chemical-induced developmental defects, such as those caused by caffeine and dexamethasone [115]. Maternal use of APAP has been shown to disrupt the development of articular cartilage in mice. This occurs through decreased mRNA expression of cartilage matrix synthesis genes (*Col2a1* and *Acan*) and suppression of extracellular matrix signaling pathways, particularly transforming growth factor beta (Tgfβ). Tgfβ is essential for cartilage development, and its suppression has been correlated with decreased fetal chondrocyte proliferation and extracellular matrix content, both of which predispose individuals to osteoarthritis in adulthood. Importantly, Tgfβ has also been implicated in the neurodevelopmental disruptions observed in ASD, and to a lesser extent, ADHD, suggesting a broader role in embryonic tissue differentiation and signaling [115]. Additionally, APAP administration severely malformed pharyngeal arch-derived cartilages in zebrafish embryos [116]. Other chemicals, such as ethanol, perchlorate, atrazine, etc., altered or reduced craniofacial cartilage, but the severe defects in this case are similar to those of endocrine disruptors, such as di-butyl phthalate and estradiol. Researchers posit that this underdevelopment of type II collagen fibers is due to apoptosis. Given the models and the prevalence of connective tissue abnormalities in these populations, further research should be conducted to discern the cause of hypermobility in ASD and ADHD individuals.

## 7. Concluding Remarks

This paper has reviewed the possible contribution of acetaminophen to the development of autism and ADHD. Much research has already been devoted to this topic, including a multitude of longitudinal studies and murine models. Nonetheless, this review expands on a theoretical framework of acetaminophen-induced ASD and ADHD grounded in a bioenergetics perspective. Upon evaluating NAPQI’s potency against mitochondria as well as the mitochondrial abnormalities present in these disorders, we recommend reviewing maternal usage of acetaminophen. While numerous studies have explored mitochondrial-targeted therapies for ASD and ADHD, including interventions aimed at improving mitochondrial function and reducing oxidative stress, a detailed discussion of these therapeutic approaches is beyond the scope of this manuscript. Future research should investigate the impact of maternal usage of APAP on offsprings’ mitochondria compared to the mitochondria of ASD/ADHD cells. Although animal and cellular models have provided valuable mechanistic insight, their translation to human neurodevelopment remains inherently limited, underscoring the need for continued clinical research. After an appeal in November 2024, the courts are still considering the Tylenol-autism/ADHD lawsuit at the time of the drafting of this article [2]. Hopefully, future research can shed light on this complex issue and work to protect the precious unborn.

## Figures and Tables

**Table 1:** Major human and murine studies linking prenatal acetaminophen exposure and ASD/ADHD. Arrows indicate an increase (↑) or decrease (↓) in incidence.

Study Type	Authors/Year	Population/Model	Exposure Measurement	Key Findings	Notes/Limitations
**Human**—**Longitudinal**	Ji et al., 2019 [3]	996 mother-child dyads	Cord blood APAP and metabolite levels	High exposure group: 3.6× ↑ ASD, 2.9× ↑ ADHD risk	Controlled for stress, BMI, age, etc.
**Murine**—**Behavioral**	Philippot et al., 2017 [25]	Mice exposed postnatally	Neonatal APAP injection	↓ Spontaneous behavior; ↓ habituation	Shows no appreciable difference between sexes
**Human**—**Longitudinal**	Baker et al., 2020 [27]	345 children (Canadian cohort)	Meconium APAP content + MRI analysis	↑ ADHD diagnosis; ↓ connectivity in key brain networks	Only 48 MRI participants; ongoing data collection
**Human**—**Sibling Study**	Ahlqvist et al., 2024 [29]	~2.5 million (Swedish cohort)	Prescription records during pregnancy	Weak association: sibling control showed no causal link	Did not capture OTC APAP; poor dose quantification
**Human**—**Sibling Study**	Gustavson et al., 2021 [35]	9820 sibling pairs (Norwegian cohort)	Self-reported usage during pregnancy	Introduced potential family factors regarding ASD/ADHD development	Self-reporting and adherence limited accuracy
**Human**—**Sibling Study**	Brandlistuen et al., 2013 [36]	2919 sibling pairs (Norwegian cohort)	Self-reported usage during pregnancy	Delayed development associated with high acetaminophen usage	Contradicts data of similar study completed with same cohort
**Murine**—**Biochemical**	Baker et al., 2023 [37]	Neonatal APAP-exposed mice	Gene expression in prefrontal cortex	↑ DNA damage and oxidative stress genes; ↓ BDNF in frontal cortex	Confirms mitochondrial dysfunction and neuroinflammation

## Abbreviations

The following abbreviations are used in this manuscript:

CDC	Centers for Disease Control and Prevention
ADHD	Attention-Deficit/Hyperactivity Disorder
ASD	Autism Spectrum Disorder
AIDS	Acquired Immunodeficiency Syndrome
APAP	Acetaminophen
NSAID	Non-steroidal Anti-inflammatory Drugs
FDA	Food and Drug Administration
SMFM	Society for Maternal Fetal Medicine
BDNF	Brain-derived Neurotrophic Factor
NAPQI	N-acetyl-p-benzoquinone imine
ROS	Reactive Oxygen Species
mtDNA	Mitochondrial DNA
nDNA	Nuclear DNA
NMDA	N-methyl-D-aspartate
Tgfβ	Transforming Growth Factor Beta
